# STEP to blood pressure management of elderly hypertension: evidence from Asia

**DOI:** 10.1038/s41440-022-00875-7

**Published:** 2022-03-11

**Authors:** Wei-li Zhang, Jun Cai

**Affiliations:** State Key Laboratory of Cardiovascular Disease, Hypertension Center, FuWai Hospital, National Center for Cardiovascular Diseases, Peking Union Medical College & Chinese Academy of Medical Sciences, Beilishi Road 167, Xicheng District, 100037 Beijing, China

**Keywords:** Aging, Hypertension, Asians, Blood pressure lowering, Cardiovascular diseases

## Abstract

With a rapidly aging population, adequate blood pressure (BP) control is critical for hypertension management and prevention of cardiovascular events. Impressive cardiovascular benefits have been observed with intensive BP control (SBP target, <120 mmHg) in the SPRINT (Systolic Blood Pressure Intervention Trial) study, even in patients 75 years of age or older. A most recent meta-analysis including 51 randomized trials with over 350,000 participants from the BPLTTC (The Blood Pressure Lowering Treatment Trialists’ Collaboration) showed that BP lowering is effective in older people for reducing major cardiovascular events. The STEP (Strategy of Blood Pressure Intervention in the Elderly Hypertensive Patients) study—a multicenter, randomized, controlled trial conducted in China, provided important evidence that intensive BP treatment (SBP target, 110 mmHg to <130 mmHg) benefits older hypertensive patients (aged 60–80 years) and reduced the incidence of cardiovascular events than standard treatment (target 130 mmHg to <150 mmHg). Because Asian people have a higher burden of hypertension and stroke than Caucasian people, intensive BP treatment has more advantages in reducing the risk of cardiovascular events including stroke in Asian hypertensive patients than in Caucasian people. Home BP monitoring is helpful to facilitate hypertension management for older patients. It should also be noted that clinical decision-making should be on a patient basis, such as fragility, diabetes, stroke, and other comorbidities, with tailored BP targets. Here we review the important clinical trials of BP control in elderly hypertension, interpretate the main findings of STEP, and also discuss the perspectives of managing hypertension in Asia.

## Introduction

Hypertension is a global public health burden and a major cause of morbidity and mortality worldwide and in Asia, but it remains inadequately controlled [1]. Several large-scale epidemiological surveys in China have showed a high prevalence of hypertension ranging from 23.2 to 44.7% whereas the low control rate from 5.6 to 15% [2–4]. The prevalence of hypertension is similarly high in other Asian countries, for example, 30% in the Republic of Korea and 47% in Mongolia [5]. More than 50% in older people over 60 years of age has hypertension. With a rapidly aging population, hypertension management among the elderly has been increasing discussed.

Systolic blood pressure (SBP) is well-recognized to be more important than diastolic blood pressure (DBP) as an independent risk predictor for cardiovascular diseases [6]. The appropriate target for SBP to reduce cardiovascular risk in older patients with hypertension remains debated. Impressive cardiovascular benefits have been observed with intensive BP control (SBP target, <120 mmHg), as compared with standard BP control (SBP target, <140 mmHg), in the SPRINT (Systolic Blood Pressure Intervention Trial), even in patients 75 years of age or older [7, 8]. A most recent meta-analysis including 51 randomized trials with over 350,000 participants from the BPLTTC (The Blood Pressure Lowering Treatment Trialists’ Collaboration) showed that BP lowering is effective in older people for reducing major cardiovascular events, down to below 120/70 mmHg [9]. The STEP (Strategy of Blood Pressure Intervention in the Elderly Hypertensive Patients) study—a multicenter, randomized, controlled trial conducted in China, provided new direct evidence that intensive BP treatment (SBP target, 110 mmHg to <130 mmHg) benefits older hypertensive patients (aged 60-80 years) and reduces the incidence of cardiovascular events than standard treatment (target 130 mmHg to <150 mmHg) [10]. Here we review the current status of BP control in elderly hypertension, focusing on the evidence from Asia, and also discuss the challenges of managing hypertension.

Point of view

- Clinical relevance
  The STEP study showed that intensive BP treatment targeting SBP < 130 mmHg markedly reduced the incidence of cardiovascular events in older hypertensive patients aged 60-80 years.
- Future direction
  The cardiovascular benefits and risks of intensive BP treatment need to be clarified in future studies for the elderly hypertensive patients with comorbidities such as prior stroke, diabetes mellitus, heart failure, and renal impairments, or aged over 80 years old.
- Consideration for the Asian population
  Because Asian people have a higher burden of hypertension and stroke than Caucasian people, intensive BP treatment has more advantages in reducing the risk of cardiovascular events including stroke in Asian hypertensive patients than in Caucasian people.

## Asia evidence on cadiovascular benefits of BP control

A number of clinical trials conducted in Asian patients have showed that lowering SBP is associated with reduced cardiovascular risk. The Systolic Hypertension in China (Syst-China) trial investigated the effects of antihypertensive treatment on cardiovascular risk in 2394 Chinese hypertensive patients aged 69–79 years with an averaged BP of 170.5/86 mmHg at baseline [11]. After a median follow-up of 3 years, step-wise active treatment with a calcium channel blocker (CCB) with or without an angiotensin-converting enzyme inhibitor (ACEI) and/or a thiazide diuretic significantly reduced the rate of stroke by 38% and cardiovascular events by 37%, with an averaged BP level reaching 150.5/81 mmHg. Another randomized controlled trial from the Felodipine Event Reduction (FEVER) in China, including 9711 Chinese hypertensive patients aged 50–79 years, showed that cardiovascular outcomes were significantly reduced by more intense therapy (low-dose hydrochlorothiazide and low-dose felodipine) achieving a mean SBP of 138 mmHg compared with less-intense therapy (low-dose hydrochlorothiazide and placebo) achieving a mean of 142 mmHg during a follow-up of 3.3 years [12]. Moreover, a subgroup analysis in patients aged ≥65 years demonstrated that a SBP goal <140 mmHg significantly reduced the risk of 44% stroke, 47% cardiovascular events, and 36% all-cause death [13].

Other studies have investigated whether the target for SBP control influences the impact of treatment on cardiovascular events. In the Valsartan in Elderly Isolated Systolic Hypertension (VALISH) trial conducted in 3260 Japanese patients aged 70–84 years with isolated systolic hypertension, SBP was reduced by 5.6 mmHg in the strict (<140 mmHg) vs. moderate (140–150 mmHg) SBP control groups (*P* < 0.001) during a median follow-up of 3 years, but this was not accompanied by a significant decrease in the cardiovascular events and renal failure [14]. Similar findings were reported in the Japanese Trial to Assess Optimal Systolic Blood Pressure in Elderly Hypertensive Patients (JATOS) study in which 4418 patients aged 65–85 years received CCB-based therapy [15]. Despite an achieved SBP difference around 10 mmHg between strict SBP control (target <140 mmHg) and mild control groups (target 140–160 mmHg), there was no difference in the incidence of cardiovascular disease and renal failure.

## STEP trial results in older patients

In the STEP trial [10], Weili Zhang and colleagues enrolled 8511 patients with hypertension aged 60-80 years to evaluate whether intensive BP-lowering treatment (SBP target of 110 to <130 mmHg) led to greater reductions in the risk of cardiovascular events than standard treatment (SBP target 130 to <150 mmHg). Total 42 clinical centers in China participated in this study, and different patient care settings were included, such as hospital-based medical centers and community-based medical centers. The primary outcome was a composite of acute coronary syndrome, stroke, acute decompensated heart failure, coronary revascularization, atrial fibrillation, or death from cardiovascular causes.

During a median 3.34-year follow-up period, the mean SBP was 126.7 mmHg in the intensive-treatment group and 135.9 mmHg in the standard-treatment group, with an average between-group difference of 9.2 mmHg; the mean DBP was 76.4 mmHg and 79.2 mmHg, respectively. Intensive-treatment resulted in a lower rate of primary-outcome events than the standard treatment (3.5% [1.0% per year] versus 4.6% [1.4% per year]), reducing the risk by 26%, and also reducing the risk of most of the individual outcomes, including stroke and acute coronary syndrome reduced by 33%. The mean number of antihypertensive medications administered per patient was 1.9 in the intensive-treatment group and 1.5 in the standard-treatment group.

STEP study is another milestone in the history of establishing active antihypertensive therapy to prevent cardiovascular diseases. Strengths of the STEP trial included the large sample size, the diverse patient population with coexisting chronic diseases including diabetic mellitus, the high rate of follow-up (97.3%), and the use of home BP monitoring.

**Fig. 1:**
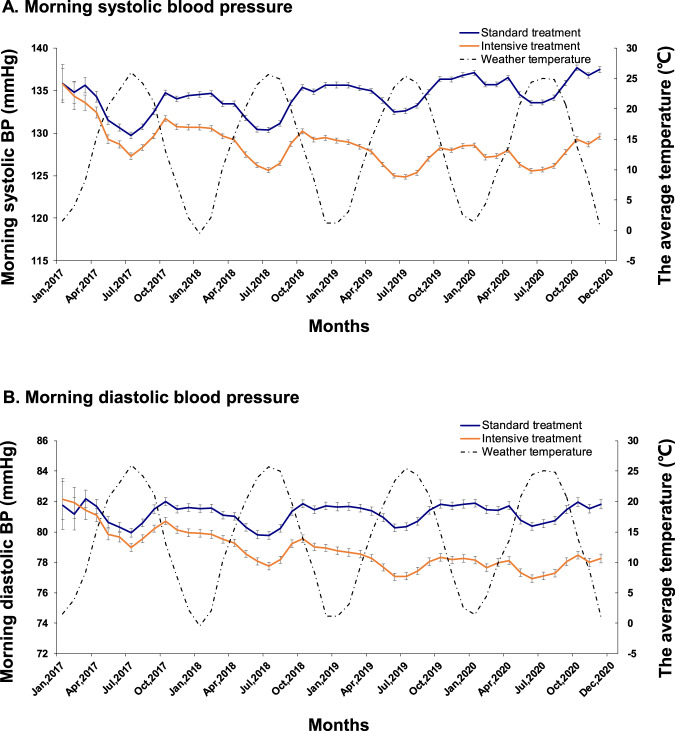
Home morning blood pressure in relation to weather temperature in the two treatment groups throughout the STEP trial. Shown are the mean morning systolic blood pressure (**A**) and diastolic blood pressure (**B**) by the treatment group. The intensive-treatment group: targeting 110 mmHg to <130 mmHg, and the standard-treatment group: targeting 130 mmHg to <150 mmHg. Reprinted with permission from Zhang et al. [10]

Of note, the STEP study used a smartphone-based application (app) to examine home BP as an adjunct to office blood pressure during the follow-up period. Every patient was provided with a validated automated home blood-pressure monitor (Omron Healthcare) and required to obtain home BP readings at least 1 day per week during follow-up. The monitor’s Bluetooth function enabled patients to upload readings to a data center via the app. The percentage of patients who used the app to transmit home BP readings was 95.8%; the remaining patients did not use the app during the follow-up period. Overall, the between-group differences in BP were significant, persistent, and consistent across the office and home BP measurements. Throughout the trial, the mean home morning SBP was 129.6 mmHg in the intensive-treatment group and 137.5 mmHg in the standard-treatment group, with an average between-group difference of 7.9 mmHg; the mean home morning DBP was 78.3 mmHg and 81.8 mmHg, respectively (Fig. 1). In addition, a seasonal variation in home BP was observed and manifested as increased BP level in winter and reduced BP level in summer.

The optimum home BP for a hypertensive patient has been usually defined as a value that is 5 mmHg less than the office BP if the office BP is already <140/90 mmHg, and therefore, 125/75 mmHg as a target home BP value will correspond to 130/80 mmHg as the target office BP value. However, sufficient evidence is needed to support this definition, particularly in aging population. The increased aortic stiffness and decreased aortic compliance have been shown to increase with duration of hypertension, and the age-related arterial stiffeness is difficult to reverse with available antihypertensive medications. In the STEP trial, observed time-varying difference between home and office BP values indicated that time-stratified cutoff values remain important for evaluation.

## Intensive BP management: from SPRINT to STEP

SPRINT were mainly conducted in hypertensive patients in Western countries, where the lifestyle and pattern of cardiovascular disease are significantly different from China. For example, the incidence and mortality of stroke in China are higher than in Western countries, while the incidence of coronary heart disease is higher in Western countries. Thus, the STEP study was in essence to investigate the veracity of the findings of the previous trial SPRINT and emphasizes the significant cardiovascular benefits of an intensive blood pressure reduction strategy in older patients with hypertension in China.

### Older hypertensive patients, but without strokes

The participants in the STEP study are relatively uncomplicated, healthy patients aged 60–80 years with hypertension. The mean age was 66.2 years, and 24% were aged 70–80 years. The mean body mass index was about 25.5 kg/m^2^. A total of 19.1% of the patients had a history of diabetes mellitus, 6.3% had a history of cardiovascular disease, 36% had hyperlipidemia, and 2.4% had chronic kidney disease. The mean Framingham Risk Score was about 19% (i.e., they had a 19% risk of having a cardiovascular event within 10 years), and 64.8% of the pateints had a Framingham risk score of 15% or higher. However, the Framingham Risk Score was formulated primarily in White populations and may overestimate the risk of cardiovascular disease in Chinese adults.

SPRINT excluded persons with diabetes mellitus, whereas the STEP trial did not. A subgroup analysis of the STEP showed a consistently beneficial, although nonsignificant, effect of intensive treatment in patients with diabetes mellitus. Of note, the statistics were underpowered, as in the Action to Control Cardiovascular Risk in Diabetes-Blood Pressure (ACCORD-BP) trial [16], which found no benefit in lowering SBP to <120 mmHg compared with <140 mmHg in terms of the trial’s primary composite cardiovascular outcomes. However, the intensively treated group in STEP and ACCORD-BP trials did have a benefit in terms of fewer stroke events. The interaction between blood-pressure treatment and glycemic control might play a significant role and warrant further investigation [17]. Thus far, robust evidence is scant, which prevents concrete conclusions regarding an appropriate BP target in patients with diabetes mellitus.

Both STEP and SPRINT trials excluded persons with a history of stroke. The Secondary Prevention of Small Subcortical Strokes (SPS3) trial found no significant benefit in lowering SBP to <130 mmHg compared with <150 mmHg for overall risk of another stroke, but a significant benefit was noted in reduced risk of intracerebral hemorrhage [18]. Further trials could assess the cardiovascular benefits of intensive BP treatment in persons with prior stroke.

### Effects of intensive BP control on mortality

In the STEP trial, there was a beneficial, although nonsignificant, tendency on the risk of death from cardiovascular causes (hazard ratio, 0.72; 95% CI, 0.39 to 1.32) in the intensive-treatment group, which might be explained by an inadequate statistic power; no effect on the risk of death from any cause was observed (hazard ratio, 1.11; 95% CI, 0.78 to 1.56) [10]. In contrast, intensive treatment in SPRINT led to significantly reduced risks of death from cardiovascular causes (hazard ratio, 0.58; 95% CI, 0.39 to 0.84) and death from any cause (hazard ratio, 0.75; 95% CI, 0.61 to 0.92) [6]. The difference might be partially explained by differences in the trial design and eligibility criteria, the SBP targets, the geographic location, along with the racial and ethnic background of the trial population.

### BP-achieved level in STEP and SPRINT

The STEP and SPRINT studies set the intensive-treatment targets at <130 mmHg and <120 mmHg, respectively. During the 3.34-year follow-up period, in the intensive-treatment group of STEP study, the percentage of patients who reached the SBP target (110 to <130 mmHg) was 67.2% at 1 year of follow-up, 70.4% at 2 years, and 77.2% at 3 years [10]. In SPRINT, more than half the participants had a SBP above the 120 mmHg target, with a mean SBP of 121.5 mmHg in the intensive-treatment group and 134.6 mmHg in the standard-treatment group.

People may ask, did SPRINT go lower? The STEP trial used the conventional way in outpatient clinic setting and measured by trained physicians or nurses. The same validated office BP measurement device (the Omron HBP-1100 U) was used for all collaborating clinical centers in STEP, which may minimizes the potential investigator bias in the determination of blood pressure. The SPRINT used a particular methodology that many felt resulted in lower than usual clinic BP measurement. The office BP was measured with the use of an automated device when the trial staff was not present when the measurement was taken, which is known to reduce the “white coat” effect. However, in clinical practice automated devices may not be available and a strict protocol for correct measurement may not be followed, and thus BP may be overestimated.

Drawz et al. evaluated the concordance between BPs obtained in routine clinical practice and those obtained using the SPRINT protocol, in a prognostic study of 3074 participants with 3 or more outpatient and trial BP measurements linking electronic health record (EHR) data [19]. The outpatient BPs measured in routine clinical practice were generally higher than BP measurements taken in SPRINT, with greater mean SBP differences apparent in the intensive treatment group (7.3 mmHg [95%CI, 7.0–7.6 mmHg]); and the difference between BP recorded in EHR and trial BP values varied widely across the clinical sites. These data indicated the potential shortcomings of comparing trial-measured BP with BP measurements from clinical EHRs. In a certain way, the BP levels achieved in the STEP study in China and in the SPRINT study using the conventional way of measuring blood pressure are probably similar.

### Several considerations related to the intensive BP control

Systolic hypertension is the predominant form of hypertension in patients aged over 50–60 years, whereas DBP tends to decrease because of age-related changes in arterial vasculature, leading to a pulse pressure widening (the difference between SBP and DBP). Given that complicated comorbidities in elderly patients, the BP lowering is a challenge. There are several considerations during the target BP management.

First, a number of observational studies indicate that lower SBP induced coronary arterial or cerebral arterial hypoperfusion, leading to increased risk of stroke [20] or cardiac ischemia [21], especially among older patients. Given that the issue of a J-shaped relationship between DBP and cardiovascular risk, the STEP study performed a sensitivity analysis. The data demonstrated that intensive treatment did not adversely affect these patients, and hazards ratio for the primary composite cardiovascular envets was respectively 0.77 (95% CI, 0.21–2.76) in patients with a DBP < 60 mmHg, 0.78 (95%CI, 0.59-1.02) in patients with a pulse pressure >60 mmHg, and 0.69 (95%CI, 0.18–2.74) in patiens with both [10].

Second, are more adverse events an acceptable trade-off? In the STEP study, the incidences of dizziness, syncope, and fracture and the results for renal outcomes did not differ significantly between the two treatment groups, nor did the incidences of angioedema, headache, cough, and hives. However, the incidence of hypotension (defined as SBP < 110 mmHg or DBP < 50 mmHg) was more common in the intensive-treatment group than in the standard-treatment group (3.4% vs. 2.6%, *P* = 0.03). Primary outcome risk was compared between the hypotension and non-hypotension group. In the whole patient population (8511), there were 14 (5.4%) in the hypotension group and 329 (4.0%) in the non-hypotension group having primary composite outcome, respectively, and the hazard ratio was 1.36 (95% CI, 0.79–2.33). It should be mentioned that patients enrolled in clinical trials tend to be less fragile and biologically active than patients seen in clinical practice; thus, the rate of adverse events reported in the trial may be lower than one would see in the real world.

Third, another concern is whether intensive treatment will worsen the cognitive function or dementia, given that cerebral hypoperfusion in small vessel disease is thought to be a major risk factor for dementia. A sub‐analysis of the SPRINT study showed that intensive treatment did not significantly reduce the risk of probable dementia compared with standard treatment, but it significantly reduced the risk of mild cognitive impairment (hazard ratio 0.81; 95%CI, 0.69–0.95) [22]. However, when compared the changes in cerebral white matter lesions and brain volume at baseline and follow‐up assessed by brain magnetic resonance imaging (MRI) in a subpopulation of SPRINT (670 patients, mean age 67.3 years), the intensive‐treatment group had a smaller increase in cerebral white matter lesions and a greater decrease in total brain volumes compared with the standard‐treatment group, although the differences in absolute values were slight [23]. A Japanese study in 55 untreated elderly hypertensive patients (average age 72.7 years) showed that increased BP pattern was negatively associated with the brain matter volume assessed by brain MRI [24]. A comparison of the effects of SBP targets on changes in cognitive function in the STEP trial is planned. Thus, the association between intensive BP treatment and cognitive function/dementia requires further investigations.

## Current guidelines on BP-lowering goals

Current hypertension guidelines have recommended the appropriate BP-lowering strategy to reduce the risk of cardiovascular events in older patients. The target is 130–139 mmHg in the European guideline [25] and <130 mmHg in the American College of Cardiology-American Heart Association guideline [26].

The major features and themes of the Asian guidelines are similar to European and US guidelines, but there are several subtle differences. For example, the 2018 Chinese Hypertension Guideline recommended that SBP should be targeted to <150 mmHg, and further <140 mmHg, if tolerated; in elderly patients aged over 80 years old, a SBP target of <150 mmHg is recommended [27]. The Japanese Society of Hypertension Guidelines for the Management of Hypertension (JSH 2019) recommends that the office BP goals for most hypertensive adults with comorbidities are at <130/80 mmHg, and for the patients over 75 years old, the optimal office BP target is <140/90 mmHg, if tolerated [28, 29]. In the 2018 Korean Society of Hypertension Guidelines for the Management of Hypertension, a target BP of <130/80 mmHg is recommended for high-risk patients with 10-year CVD risk of >15% or for patients with cardioasular disease over 50 years old, and in contrast, a target BP of <140/90 mmHg is recommended for hypertensive patients at low-to-moderate risk [30].

## Clinical challenges of elderly hypertension in Asia

Lower BP control is important in not only Western population but also Asian population. Althouth the awareness, treatment, and control rates of hypertension are generally improving, BP control remains inadequate worldwide and in Asia. Reasons for poor BP control include low awareness of hypertension among physicians and patients, under-treatment, and tolerability problems with antihypertensive drugs.

Approximate 50–80% of people are unaware of the hypertensive condition and/or its potentially fatal impact on cardiovascular events. The control rates (defined as below 140/90 mmHg) in all subjects with hypertension are approximate 15% in China, 40% in Japan, 41% in Korea, and 50% in USA and Europe [1, 2]. Poor knowledge about hypertension and the need for chronic therapy to achieve and maintain BP control often leads to bad adherence. In addition, physicians may also be concerned about the potentially harmful effects of excessive reduction in DBP when trying to attain SBP goal with antihypertensive drugs in older patients with hypertension.

Practical issues have therefore been raised with regard to the active BP treatment strategy. A lower BP control may prevent cardiovascular disease events in high-risk patients and reduce health care costs, as compared with standard control, but it would be more demanding to reach and may result in an increased risk of potential adverse events and higher costs, such as more frequent clinic visits, laboratory tests, and medications. Considering this point, Richman et al showed that intensive control of SBP prevented cardiovascular disease events and prolonged life span, at a cost below common willingness-to-pay thresholds [31]. Similarly, Li et al used nationally representative data from the China Health and Retirement Longitudinal Study (CHARLS) (2011-2012) to predict the medical and economic implications of this intensive SBP treatment among people meeting the SPRINT eligibility, and their data showed that intensive SBP control (<120 mmHg) is cost-effective in preventing cardiovascular events and obtaing gains in life-years [32]. Based on the STEP trial, several issues, such as the effects of intensive BP control (<130 mmHg) on quality of life, cost effectiveness, and long-term clinical outcomes, could be addressed in future research.

## Perspectives in Asia

Asian people have a higher burden of hypertension and stroke as well as a steeper blood pressure-vascular event relationship than Caucasian people [33]. The participants in the STEP study were all Chinese; thus, these findings are likely applicable to the Asian population. Intensive BP treatment targeting SBP < 130 mmHg could be considered for Asian elderly people with hypertension. As mentioned above, achieving the SBP goal of less than 130 mmHg may be challenging because doing so could require the use of additional medications, more careful monitoring, and more frequent clinic visits. Clinicians should engage patients in a shared decision-making process, with discussion of the benefits and risks associated with intensive lowering of blood pressure.

## In summary

Finding the best antihypertensive target is quite an important task. In our opinion, lower SBP is better if adverse events can be monitored, avoided, or managed. Certainly, it would be prudent to aim for a more conservative goal in elderly patients who are frail and at risk for falls, or in patients with a DBP of 60–65 mmHg and co-existing coronary artery disease or peripheral artery stenosis. Home BP monitoring is helpful to facilitate hypertension management for older patients. For the elderly hypertensive patients with comorbidities such as prior stroke, diabetes mellitus, heart failure, and renal impairments, or aged over 80 years old, further trials could assess the cardiovascular benefits of intensive BP treatment.

The evidence, from the SPRINT trial, STEP trial, and the individual participant-level meta-analysis in BPLTTC, demonstrates that now is an time to act to return elevated blood pressure to its status as a continuous-variable risk factor, instead of treating it as a dichotomous disease. Blood pressure should be managed as an integrated part of a patient’s risk profile [34].
